# Antibiotic susceptibility of pathogens isolated in respiratory tract samples of recently hospitalized patients

**DOI:** 10.1128/spectrum.01422-24

**Published:** 2025-02-05

**Authors:** Anat Reiner-Benaim, Oryan Henig, Pilar Coronel, Mercedes Gimeno, Gilad Rozenberg, Dima Shlon, Ami Neuberger

**Affiliations:** 1 Department of Epidemiology, Biostatistics and Community Health Sciences, Ben-Gurion University of the Negev, Beer-Sheva, Israel; 2 Infection Prevention and Control Unit, Tel Aviv Sourasky Medical Center, Tel Aviv, Israel; 3 Scientific Department, Meiji Pharma Spain, Madrid, Spain; 4 Unit of Infectious Diseases, Rambam Healthcare Campus, Haifa, Israel; 5 Department of Internal Medicine D, Division of Internal Medicine, Rambam Healthcare Campus, Haifa, Israel; 6 Bruce Rappaport Faculty of Medicine, Technion, Haifa, Israel; MultiCare Health System, Tacoma, Washington, USA

**Keywords:** lower respiratory tract infection, pneumonia, microbiology, pathogens, community acquired infections

## Abstract

Community-acquired lower respiratory tract infections (CA-LRTIs) treatment is largely empirical as microbiologic testing is rarely performed. Here, we provide microbiologic data of severe CA-LRTI cases requiring hospitalization. We aim to describe the distribution and susceptibility patterns of pathogens causing severe CA-LRTI. We analyzed respiratory samples from recently admitted patients with CA-LRTIs to assess pathogen distribution and antibiotic susceptibility patterns. We divided patients into three groups: CA-LRTI and no prior healthcare exposure, CA-LRTI with healthcare exposure, and patients diagnosed with LRTI 48 to 7 days of hospital admission. In a sub-cohort, we assessed the bacteria’s susceptibility to cefditoren. A total of 1,395, 2,212, and 2,760 samples were included in the three study groups. Gram-negative bacteria were the most common bacteria isolated. *Streptococcus pneumoniae* was over-represented in patients admitted to the intensive care unit (ICU) in the first two study groups, and was fully susceptible to penicillin in only ~50% of cases, and to fluoroquinolones and third-generation cephalosporins including cefditoren in >95% of cases. Susceptibility of Gram-negative bacteria to penicillins and to second-generation cephalosporins was lower than 50%. Age, admission to an ICU or surgical department, healthcare-associated infections, and infections with Gram-negative bacteria, *Staphylococcus aureus*, and *Stenotrophomonas*/*Acinetobacter baumannii* were associated with increased mortality. These results highlight pathogen diversity and concerning antibiotic susceptibility patterns in LRTI. These findings emphasize the importance of improving diagnostics and addressing antibiotic resistance in the effective management of LRTI.

**IMPORTANCE:**

This survey aims to describe the microbiologic aspects of community-acquired lower respiratory tract infections (CA-LRTI) in a large cohort of patients recently admitted to hospital. In a small sub-study, we assessed antibiotic susceptibility to cefditoren, an oral third-generation cephalosporin not used in Israel. By analyzing specimens from recently admitted patients with CA-LRTI, we aim to provide physicians with the relevant microbiologic data of the more severe CA-LRTI cases, i.e., those that resulted in hospital admission. Such microbiological data would provide primary care and emergency room physicians with additional insights as to the causative agents of severe CA-LRTI.

## INTRODUCTION

Infections involving the lower respiratory tract play a major role in morbidity and mortality rates, and in socio-economic costs worldwide (1), being among the top 10 causes of death worldwide, and the leading cause among communicable diseases (2). There is a wide variety of diseases included in the community-acquired lower respiratory tract infections (CA-LRTIs) with different causative pathogens (3), and current treatment is essentially empirical, often resulting in an inaccurate use of antibiotics (4). The use of antibiotic treatment without certain knowledge of the causative pathogen can lead to treatment failure and even the development of resistances (5). As a result, along with the threatening growth of antibiotic resistance, a decrease in the patient’s health status, an increase in the likelihood of hospitalization, and a raise in treatment costs may occur (6). Respiratory pathogens in Israel are frequently resistant to many oral antibiotics, but still susceptible to third-generation cephalosporins and fluoroquinolones (7). However, the use of fluoroquinolones is associated with adverse effects such as QT interval prolongation, rupture of aortic abdominal aneurysms, and tendinopathies (8, 9), and with the development of resistances (10). This scenario brings up the need to optimize the use of antibiotics for community-acquired LRTI. For drug-resistant pathogens, the use of third-generation oral cephalosporins, such as cefditoren, may be beneficial (11, 12), improving health outcomes and costs and curbing antibiotic resistance. Excessive use, on the other hand, should be discouraged.

In the few studies that have examined the microbiology of community-acquired pneumonia among relatively young patients, the only culturable pathogens that were found in a significant proportion of cases (i.e., >5%) are *S. pneumoniae* and *Haemophilus influenzae* (13). Other pathogens, such as *Moraxella* spp., *Bordetella* spp., and *Staphylococcus aureus,* are uncommon (14). In contrats, common pathogens such as *Legionella pneumophila*, *Mycoplasma pneumoniae,* or *Chlamydia pneumoniae* are only diagnosed through molecular methods or serological tests (15, 16). Gram-negative bacteria are detected in 2.8%–14% of community-acquired pneumonia cases, particularly in the elderly and patients who are admitted from a long-term care facility (LTCF) (17–19).

## MATERIALS AND METHODS

### Epidemiological and susceptibility cohort of patients with CA-LRTI

A retrospective observational cohort study was performed at Rambam Health Care Campus (RHCC), a 970-bed primary and tertiary care hospital in Haifa, Israel.

The main cohort included all consecutive respiratory tract samples obtained from patients older than 12 years who were admitted to the RHCC between January 2014 and January 2020. Patients were identified through laboratory reports, where consecutive respiratory samples collected from hospitalized patients were screened. All patients providing consecutive sputum samples were considered to have LRTI. In accordance with hospital guidelines, respiratory tract specimens are obtained only from patients with clinical infection. To ensure that included cases include patients with actual infection, we analyzed 50 random patients’ files to screen whether these guidelines were indeed followed.

The cohort was defined according to the following criteria: (i) the time elapsed from date of admission to the date the sample was collected; (ii) whether the admission was from a LTCF; and (iii) if the patient had been hospitalized within the past 3 months. Accordingly, the study consisted of three cohorts as follows: (i) CA-LRTI with no known healthcare exposure: cultures which were obtained within less than 48 hours of admission of patients who were not residing in an LTCF and were not hospitalized in the RHCC within the past 3 months; (ii) CA-LRTI with recent hospital admission or residence in an LTCF (HCA-LRTI): cultures which were collected within 48 hours of admission of patients who were either admitted from an LTCF or hospitalized in the RHCC within the past 3 months; (iii) LRTI diagnosed 48 hours to 7 days within admission (HA-LRTI): cultures which were collected from patients who were hospitalized for no less than 3 days but for no more than 7 days.

Bacterial pathogens were divided into seven groups: (i) Enterobacteriaceae; (ii) Gram-negative bacteria associated with community-acquired infections*—Haemophilus influenzae*, *Moraxella* and *Bordetella* spp.; (iii) non-fermenting Gram-negative bacteria*—Pseudomonas* and *Burkholderia* spp.; (iv) other Gram-negative bacteria*—Serratia, Providencia,* and *Proteus* spp.; (v) *S. aureus*; (vi) opportunistic bacteria common among intensive care unit (ICU) patients*—Stenotrophomonas* and *Acinetobacter* spp.; and (vii) *S. pneumoniae*. We defined a group of pathogens as “classic community pathogens” based on previous large studies that showed *S. pneumoniae* and *H. influenzae* to be by far the most common bacterial pathogens to cause CA-LRTI (12). *Moraxella* and *Bordetella* spp., although uncommon, were also included in this group as they rarely cause hospital-acquired pulmonary infections (20, 21).

### Susceptibility to cefditoren

An additional cohort of patients was used to assess bacterial susceptibility to cefditoren. Bacterial samples from the respiratory tract were taken from 163 patients recently admitted to RHCC between 1 October 2020 and 6 June 2021. Samples included sputum, broncho-alveolar lavage, or tracheal aspirates from intubated patients. Inclusion criteria were clinical LRTI with respiratory tract samples from patients aged 18 or older admitted within 48 hours of sample acquisition, with lower respiratory tract infections caused by bacteria potentially susceptible to third-generation cephalosporins. Clinical LRTI was defined as either an infiltrate on chest X-ray or computed tomography (CT), or two of the following symptoms and signs: fever, cough, dyspnea, or hypoxemia. Exclusion criteria we re samples obtained without clinical infection, samples collected more than 48 hours after admission, or samples containing bacteria inherently resistant to third-generation cephalosporins. (e.g., *Pseudomonas aeruginosa*).

Bacterial susceptibilities were described for four groups of pathogens: *S. pneumoniae* (group 1), *H. influenzae* and other *Haemophilus* spp. (group 2), other Gram-negative bacteria including *Escherichia coli*, *Klebsiella* spp., *Enterobacter* spp., *Serratia marcescens, Proteus mirabilis, Citrobacter* spp., *Serratia* spp., *Providencia stuartii,* and *Morganella morgani* (group 3), and *S. aureus* (group 4).

#### Data collection

For each patient’s admission throughout the study period, all unique bacterial species were included. Data pertaining to demographics, source of admission (home, LTCF), hospitalization at the RHCC within the past 90 days, Charlson Comorbidity Index (CCI, not age-adjusted), active malignancy, and admission unit were collected with the use of the institution’s electronic medical records for each admission. Microbiology data included dates of sample collection, dates of results, name of bacteria species, and antimicrobial susceptibility.

#### Susceptibility assessment

Drug resistance was defined according to the Clinical and Laboratory Standards Institute breakpoints for susceptibility. Bacteria with intermediate drug susceptibility were defined as resistant. Susceptibility to cefditoren was performed with the use of the disk diffusion method. (22)

#### Outcomes

The main outcome measures were the microbiologic susceptibility patterns of LRTI-causative pathogens. Secondary outcomes in the main cohort included factors associated with 14-day mortality in the entire cohort, and in the subgroup of patients infected with classic community pathogens. Fourteen-day mortality was assessed in relation to the date the index culture was obtained.

#### Ethics

For both studies, demographic and clinical data were recorded from patients’ electronic files, without recording patients’ identifying data. The testing was performed retrospectively, with samples previously received in the laboratory as part of routine patient care, and did not affect patient treatment.

#### Statistical analysis

In the case of epidemiological surveillance, continuous variables were described with the use of median and 25%–75% interquartile ranges (IQRs). We analyzed factors associated with a 14-day mortality in the entire cohort, and in the cohort of patients with classic community pathogens (*S. pneumoniae, H. influenzae, Bordetella* spp.*, Moraxella* spp.) using a multivariate logistic regression with a stepwise model reduction, and accounting for age, gender, referring ward, and place of acquisition. For the study related to cefditoren susceptibility, descriptive statistics were used for antibiotic susceptibility data. Chi-square and Fisher’s exact test were used for categorical variables, as appropriate. For continuous variables Student’s *t*-test or non-parametric tests were used for normally and abnormally distributed variables, respectively. A *P*-value of 0.05 or lower was considered statistically significant.

## RESULTS

### Epidemiological and susceptibility surveillance of CA-LRTI

A total of 1,395, 2,212, and 2,760 samples were included in the CA-LRTI, HCA-LRTI, and HA-LRTI groups, respectively. Of the 2,212 HCA-LRTI samples, 295 were obtained from patients coming from LTCFs, while 1,917 were obtained from patients with previous hospital admissions. Regarding the entire cohort, samples taken from female patients accounted for 29.6%. The mean age was 60.6 ± 19.9 years (median 65, IQR = 50–75), and the mean CCI was 5.7 ± 4.1 (median 5, IQR = 2–8.5). Samples were obtained in the pediatric ward (4.2%), ICU (28.6%), emergency room and internal medicine wards (50.1%), oncology and hemato-oncology wards (2.5%), and surgery and orthopedics wards (14.1%). Sputum samples, tracheal aspirations. or broncho-alveolar lavage was used in 61.4%, 29.5%, and 9% of all specimens, respectively. These data are presented in Table 1.

**TABLE 1:** Characteristics of 6,367 patients with lower respiratory tract infections

Age (median, IQR)	65 (50,75)
Female (*N* [%])	1,884 (30)
Charlson Comorbidity Index (median, IQR)	5 (2, 8.5)
Place and timing of acquisition	
Community with no healthcare exposure (*N* [%])	1,395 (21.9)
Community with healthcare exposure (*N* [%])	2,212 (34.7)
Cultures obtained 48 hours–2 days within admission (*N* [%])	2,760 (43.3)
Department	
Pediatric (*N* [%])	267 (4.2)
Internal medicine (*N* [%])	3,190 (50.1)
Surgery-orthopedic (*N* [%])	897 (14.1)
Hemato-oncology (*N* [%])	190 (2.5)
ICU (*N* [%])	1,823 (28.6)
Source of specimen	
Sputum (*N* [%])	3,912 (61.4)
Tracheal aspiration (*N* [%])	1,879 (29.5)
Broncho-alveolar lavage (*N* [%])	576 (9)

Pathogen distribution among the three study cohorts is shown in Table 2. The probability of isolating bacteria which we initially expected to be “classic” community-acquired pathogens, namely *S. pneumoniae*, *H. influenzae*, *Bordetella* spp., and *Moraxella* spp., was higher among patients with CA-LRTI (*P* < 0.0001 for all comparisons). Enterobacteriaceae, non-fermenting Gram negative bacteria, and other Gram-negative bacteria were more frequently isolated from older patients with multiple comorbidities with HCA-LRTI and HA-LRTI and were the most common pathogens isolated in all cohorts (*P* < 0.0001 for all comparisons), while *S. aureus* was relatively equally distributed among the three study cohorts. These observations were also true when patients were divided into age groups (12–18 years; >18–40 years; >40 years) (Fig. S1), or according to the Charlson Comorbidity Index (CCI = 0; 1 ≤ CCI ≤ 4; CCI > 4) (Fig. S2).

**TABLE 2:** Pathogen distribution among patients with community-acquired, healthcare-associated, and hospital-acquired LRTI

Bacteria	Community-acquired LRTI with no healthcare exposure Absolute number (%) (*N* = 1,395)	Community-acquired LRTI with healthcare exposure Absolute number (%) (*N* = 2,212)	LRTI diagnosed 3–7 days after admission Absolute number (%) (*N* = 2,760)
*Streptococcus pneumoniae*	176 (12.6%)	141 (6.4%)	95 (3.4%)
*Stenotrophomonas* and *Acinetobacter* spp.	69 (4.9%)	161 (7.3%)	315 (11.4%)
*Staphylococcus aureus*	227 (16.3%)	354 (16.0%)	407 (14.8%)
*Serratia, Providencia*, and *Proteus* spp.	66 (4.7%)	173 (7.8%)	225 (8.2%)
*Pseudomonas* and *Burkholderia* spp.	210 (15.1%)	539 (24.4%)	556 (22.1%)
*Haemophilus* spp., *Bordetella* spp., *Moraxella* spp.	276 (19.8%)	280 (12.7%)	302 (10.9%)
*Citrobacter* spp.*, Enterobacter* spp*, Escherichia coli, Klebsiella* spp.	371 (26.6%)	564 (25.5%)	860 (31.2%)

^*a*^ LRTI, lower respiratory tract infection. Community-acquired LRTIs consist of samples taken within 0–2 days of hospital admission. Healthcare-associated LRTIs consist of samples taken within 0–2 days of hospital admission and with recent healthcare exposure. Hospital-acquired LRTIs consist of samples taken within 3–7 days of hospital admission.

The relative proportion of *S. pneumoniae* was significantly higher (*P* < 0.001) among patients aged 18 to 40 years old (9.8% of all samples) when compared to younger patients (7.1%), or to adults aged more than 40 years old (5.9%). However, in absolute numbers, most cases occurred in patients older than 40 years. Similarly, the proportion of samples with *H. influenzae*, *Moraxella* spp., and *Bordetella* spp. were 15.1%, 18.1%, and 12.6% of samples from patients in the three respective age groups (*P* < 0.001 for all comparisons), but in absolute numbers, the vast majority of samples were acquired from patients older than 40 years.

Pathogen distribution in samples taken from patients in the ICU is shown in Table 3. Among all patients with LRTI, *S. pneumoniae* was over-represented among patients with CA-LRTI admitted to the ICU (accounting for 12.6% of all samples, but 20% of samples admitted to the ICU), and among patients with HCA-LRTI (accounting for 6.4% of all patients, but 10.1% among patients admitted to the ICU, *P* < 0.001 for both comparisons). Other classic community pathogens (*H. influenzae, Moraxella* spp., and *Bordetella* spp.), Gram-negative bacteria, and *S. aureus* were not more prevalent among patients in the ICU when compared to their relative frequency in the entire cohort.

**TABLE 3:** Frequency of pathogens in respiratory tract cultures from hospitalized patients

Entire cohort *N* = 6,367				ICU *N* = 1,823			
	Community-acquired LRTI (no healthcare exposure) *N* = 1,395	Community-acquired LRTI (with healthcare exposure) *N* = 2,212	LRTI diagnosed 3–7 days after admission *N* = 2,760		Community-acquired LRTI (no healthcare exposure) *N* = 454	Community-acquired LRTI (with healthcare exposure) *N* = 426	LRTI diagnosed 3–7 days after admission *N* = 943
*Streptococcus pneumoniae* *N* (%)	176 (12.6)	141 (6.4)	95 (3.4)	*Streptococcus pneumoniae* *N* (%)	91(20)	43 (10.1)	32 (3.4)
*Stenotrophomonas, Acinetobacter* *N* (%)	69 (4.9)	161 (7.3)	315 (11.4)	*Stenotrophomonas, Acinetobacter* *N* (%)	24 (5.3)	31 (7.3)	151 (16)
*Staphylococcus aureus* *N* (%)	227 (16.3)	354 (16)	407 (14.8)	*Staphylococcus aureus* *N* (%)	89 (18.9)	70 (16.4)	98 (10.4)
*Serratia, Providencia, Proteus* *N* (%)	66 (4.7)	173 (7.8)	225 (8.2)	*Serratia, Providencia, Proteus* *N* (%)	19 (4.2)	27 (6.3)	77 (8.2)
*Pseudomonas species, Burkholderia* *N* (%)	210 (15.1)	539 (24.4)	556 (22.1)	*Pseudomonas species, Burkholderia* *N* (%)	32 (7)	51 (12)	174 (18.5)
*Haemophilus influenzae*, *Bordetella* spp., *Moraxella* spp. *N* (%)	276 (19.8)	280 (12.7)	302 (10.2)	*Haemophilus influenzae*, *Bordetella* spp., *Moraxella* spp. *N* (%)	89 (19.6)	92 (21.6)	87 (9.2)
*Citrobacter, Enterobacter, K. pneumoniae, E. coli* *N* (%)	371 (26.6)	564 (25.5)	860 (31.2)	*Citrobacter, Enterobacter, K. pneumoniae, E. coli* *N* (%)	113 (24.9)	112 (26.3)	324 (34.3)

Antibiotic susceptibilities of various bacteria are shown in Table 4. *S. pneumoniae* was fully susceptible to penicillin in only around 50% of cases, and to erythromycin in 65% of cases. *S. pneumoniae* bacteria were susceptible in more than 95% of cases to fluoroquinolones (both ciprofloxacin and levofloxacin) and to third-generation cephalosporins (ceftriaxone). Other classic community-acquired pathogens (*H. influenzae, Moraxella* spp., and *Bordetella* spp.) were also nearly universally susceptible to fluoroquinolones and third-generation cephalosporins, but about 25% were resistant to ampicillin. Gram-negative bacteria were resistant to penicillin, penicillin and β-lactamase combinations, and second-generation cephalosporins in more than 50% of cases. Susceptibility rates to fluoroquinolones and third-generation cephalosporins were around 80% and 75%, respectively (Table 4).

**TABLE 4:** Antimicrobial susceptibilities of pathogens causing lower respiratory tract infections^*a*^

Antibiotic % Suscept.	Penicillin	Ampicillin	Amox/Clav	Oxacillin	Cefuroxime	Ceftriaxone	Cipro	Levo	Erythro	TMP/SMZ	Clindamycin	Linezolid	Vancomycin
	*Streptococcus pneumoniae*												
Community *N* = 176	59.1	–^*b*^	–		–	97.9	–	98.6	73.4	81.3	83.1	100	100
HCA *N* = 141	43.4	–	–		–	95.3		97.2	58.5	83	72.9	100	100
Hospital *N* = 95	54.5	–	–		–	97.2	–	100	62.3	78.9	–	100	100
	*Staphylococcus aureus*												
Community *N* = 227	23.6	–	–	83.5	–	–	81.1	65.6	66.5	99.6	–	100	100
HCA *N* = 354	17.4	–	–	63.9	–	–	66.3	–	56.8	–	57.3	100	99.4
Hospital *N* = 403	25.9	–	–	66	–	–	69.4	–	57.6	98.5	59.1	100	99.8
	*Citrobacter, Enterobacter, E. coli, Klebsiella*												
Community *N* = 371	–	14.9	58.4		69.2	84.3	89.9	–	–	85	–	–	–
HCA *N* = 564	–	8	47.9		49.4	68.7	77.5	–	–	71.1	–	–	–
Hospital *N* = 860	–	6.7	49.8		51.7	70.4	82.8	–	–	74	–	–	–

^*a*^ LRTI, lower respiratory tract infection. Test susceptibility, with ceftriaxone as a reference. Amox/Clav, amoxicillin and clavulonic acid. Cipro, ciprofloxacin. Levo, levofloxacin. Erythro, erythromycin.

^*b*^ “–”, means unsusceptible.

Table 5 presents the frequency of classic community pathogens, Gram-negative bacteria, and *S. aureus* among patients who died within 14 days of obtaining the index culture. *S. pneumoniae* and Gram-negative bacteria were over-represented among patients who died in the CA-LRTI group.

**TABLE 5:** Fourteen-day mortality among patients with respiratory cultures of typical community pathogens, *Staphylococcus aureus*, and Gram-negative bacillii

Community-acquired LRTI with no healthcare exposure *N* = 1,395			Community-acquired LRTI with healthcare exposure *N* = 2,212			LRTI diagnosed 3–7 days after admission *N* = 2,760		
Bacterial species	Dead (*N* = 161)	Alive (*N* = 1,234)	Bacterial species	Dead (*N* = 342)	Alive (*N* = 1,870)	Bacterial species	Dead (*N* = 551)	Alive (*N* = 2,209)
*Streptococcus pneumoniae* *N* = 181 *N* (%)	29 (18)	152 (12.3)	*Streptococcus pneumoniae* *N* = 141 *N* (%)	18 (5.3)	123 (6.6)	*Streptococcus pneumoniae* *N* = 95 *N* (%)	9 (1.6)	86 (3.9)
*Stenotrophomonas, Acinetobacter* *N* = 69 *N* (%)	6 (3.7)	63 (5.1)	*Stenotrophomonas, Acinetobacter* *N* = 161 *N* (%)	30 (8.8)	131 (7)	*Stenotrophomonas, Acinetobacter* *N* = 315 *N* (%)	83 (15.1)	232 (10.5)
*Staphylococcus aureus* *N* = 227 *N* (%)	29 (18)	198 (16)	*Staphylococcus aureus* *N* = 354 *N* (%)	62 (18.1)	292 (15.6)	*Staphylococcus aureus* *N* = 407 *N* (%)	95 (17.2)	312 (14.1)
*Serratia, Providencia, Proteus* *N* = 66 *N* (%)	8 (5)	58 (4.7)	*Serratia, Providencia, Proteus* *N* = 173 *N* (%)	26 (7.6)	147 (7.9)	*Serratia, Providencia, Proteus* *N* = 225 *N* (%)	47 (8.5)	178 (8.1)
*Pseudomonas* species, *Burkholderia* *N* = 210 *N* (%)	17 (10.6)	193 (15.6)	*Pseudomonas* species, *Burkholderia* *N* = 539 *N* (%)	54 (15.8)	485 (25.9)	*Pseudomonas* species, *Burkholderia* *N* = 556 *N* (%)	116 (21.1)	440 (19.9)
*Haemophilus influenzae*, *Bordetella* spp., *Moraxella* spp. 276 *N* (%)	26 (16.1)	250 (20.3)	*Haemophilus influenzae*, *Bordetella* spp., *Moraxella* spp. *N* = 280 *N* (%)	29 (8.5)	251 (13.4)	*Haemophilus influenzae*, *Bordetella* spp., *Moraxella* spp. *N* = 302 *N* (%)	36 (6.5)	266 (12)
*Citrobacter, Enterobacter, K. pneumoniae, E. coli* *N* = 371 *N* (%)	51 (31.7)	320 (25.9)	*Citrobacter, Enterobacter, K. pneumoniae, E. coli* *N* = 564 *N* (%)	123 (36)	441 (23.6)	*Citrobacter, Enterobacter, K. pneumoniae, E. coli* *N* = 860 *N* (%)	165 (29.9)	695 (31.5)

Factors associated with 14-day mortality were analyzed in the entire cohort, including Gram-negative bacteria and classic community pathogens (*S. pneumoniae, H. influenza, Moraxella* spp., and *Bordetella* spp.). In the multivariable analysis, factors that were associated with increased mortality were age, admission to an ICU, admission to a surgical department, HCA-LRTI and HA-LRTI (when compared to CA-LRTI), and infections with *S. aureus*, Enterobacteriaceae, or *Stenotrophomonas* and *A. baumannii* (Tables 6 and 7).

**TABLE 6:** Predictors for 14-day mortality among hospitalized patients with LRTI^*a*^

Predictor	Adjusted OR (95% CI)	*P*-value
Age^*e*^	1.026 (1.02, 1.032)	<0.001
Department		
Internal medicine	1 (reference)	
Pediatric	0.57 (0.77, 4.27)	0.59
ICU	3.37 (2.34, 4.85)	<0.001
Oncology-hematology	0.53 (0.07, 3.97)	0.53
Surgery-orthopedic	2.48 (1.34, 4.60)	0.004
Bacterial species		
*Streptococcus pneumoniae*	1 (reference)	
Gram-negative bacteria associated with community-acquired infections^*b*^	0.83 (0.57, 1.21)	0.34
*Staphylococcus aureus*	1.55 (1.10, 2.19)	0.01
Non-fermenting Gram-negative bacteria^*c*^	1.06 (0.75, 1.49)	0.75
*Stenotrophomonas* and *Acinetobacter* spp.	1.69 (1.16, 2.45)	0.006
Enterobacteriaceae	1.39 (1.001, 1.93)	0.048
Other Gram-negative bacteria^*d*^	1.23 (0.83, 1.82)	0.30
Timing and place of acquisition		
Community-acquired without healthcare exposure	1 (reference)	
Community-acquired with healthcare exposure	1.64 (1.21, 2.23)	0.002
LRTI cases diagnosed 48 hours–7 days after admission	3.08 (2.28, 4.15)	<0.001

^*a*^ OR, odds ratio; CI, confidence interval; ICU, intensive care unit; LRTI, lower respiratory tract infection.

^*b*^ *Haemophilus influenzae*, *Moraxella* and *Bordetella* spp.

^*c*^ *Pseudomonas* and *Burkholderia* spp.

^*d*^ *Serratia, Providencia,* and *Proteus* spp.

^*e*^ Increase for each year.

**TABLE 7:** Predictors for 14-day mortality among patients who had respiratory cultures with typical community pathogens^*a*^

Predictor	Adjusted OR (95% CI)	*P*-value
Age^*b*^	1.025 (0.99, 1.05)	0.056
Charlson Comorbidity Index		
Charlson Comorbidity Index = 0 (reference)	1	
Charlson Comorbidity Index 1–4	0.40 (0.14, 1.15)	0.09
Charlson Comorbidity Index > 4	0.56 (0.17, 1.84)	0.34
Department		
Internal medicine	1 (reference)	
Children	0.56 (0.17, 1.84)	0.34
ICU	2.23 (1.44, 3.55)	<0.001
Oncology-hematology	1.74 (0.63, 4.78)	0.28
Surgery-orthopedic	1.87 (1.07, 3.26)	0.03
Timing and place of acquisition		
Community acquired (no healthcare exposure)	1 (reference)	
Community acquired (with healthcare exposure)	0.88 (0.08, 9.32)	0.92
LRTI cases diagnosed 48 hours–3 days within admission	0.97 (0.20, 4.82)	0.97

^*a*^ OR, odds ratio; CI, confidence interval; ICU, intensive care unit.

^*b*^ Increase for each year.

In the multivariable analysis that included only classic community-acquired pathogens (*S. pneumoniae, H. influenzae, Moraxella* spp., and *Bordetella* spp.), factors that were associated with an increased risk of mortality included ICU and surgical ward admission. Because of a strong interaction between the place of acquisition and the CCI, the cohort was stratified into subgroups according to the place of acquisition and the multivariable analysis for a 14-day mortality was repeated. In the subgroup of HCA-LRTI, a CCI between 1 and 4 as well as a CCI greater than 4 were independent predictors for a 14-day mortality (OR 12.94, 95% CI 1.65, 101.15, *P* = 0.01; OR 14.84, 95% CI 1.70, 129.15, *P* = 0.01, respectively). The CCI was not associated with a 14-day mortality in the other subgroups.

### Cefditoren as therapeutic option in CA-LRTI

A total of 210 specimens from 163 patients were analyzed. The mean age of the patients was 63.9 years old, 74.8% were male, 63.19% had active LRTI, and 36.91% had upper respiratory tract infection. Demographic variables are detailed in Table 8.

**TABLE 8:** Demographic variables for cohort analyzed for cefditoren susceptibility

Age^*a*^	63.9 (17.2)
Male^*b*^	122 (74.80)
Active lower respiratory tract infection	
Patients^*b*^	103 (63.19)
Specimens^*b*^	136 (64.76)
No LRTI^*b*^	
Patients^*b*^	60 (36.91)
Specimens^*b*^	74 (35.24)

^*a*^ *n* (SD).

^*b*^ *n* (%).

Specimens obtained from both subpopulations (N-LRTI and LRTI) were cultured: *S. pneumoniae* grew in 22 samples, *H. influenzae* and other *Haemophilus* spp. grew in 35 samples, other Gram-negative bacteria grew in 123 samples, and *S. aureus* grew in 30 samples. Antibiotic susceptibility studies showed 86.4% and 45.5% of susceptibility of *S. pneumoniae* to ceftriaxone and penicillin-G, respectively, and 100% of susceptibility to cefditoren and ciprofloxacin. Of isolated *Haemophilus* spp., 74.3% were susceptible to ampicillin, 91.4% to ceftriaxone, 80.0% to cefuroxime, 97.1% to ciprofloxacin, and 100% to cefditoren. For other Gram-negative bacteria, susceptibility rates were 11.4% to ampicillin, 57.7% to cefuroxime, 80.5% to ceftriaxone, 69.1% to ciprofloxacin, and 79.9% to cefditoren. Finally, for *S. aureus*, susceptibility for oxacillin was 70.0% while for cefditoren, it was 80.0%. Antibiotic susceptibility data are summarized in Table 9.

**TABLE 9:** Susceptibilities of bacteria in patients with respiratory tract infections analyzed for cefditoren susceptibility

	*Streptococcus pneumoniae* (*n* = 22)	*Haemophilus influenzae* (*n* = 35)	Gram-negative bacteria (*n* = 123)	*Staphylococcus aureus* (*n* = 30)
Ampicillin		26/35 (74.3%)	14/123 (11.4%)	
Penicillin-G	10/22 (45.5%)			
Oxacillin				21/30 (70.0%)
Cefuroxime		28/35 (80.0%)	71/123 (57.7%)	
Ceftriaxone	19/22 (86.4%)	32/35 (91.4%)	99/123 (80.5%)	
Cefditoren	22/22 (100%)	35/35 100%	98/123 (79.7%)	24/30 (80.0%)
Ciprofloxacin	22/22 (100%)	34/35 (97.1%)	85/123 (69.1%)	

For those patients showing active LRTI, data on vital signs, laboratory results, and Charlson Comorbidity Index (CCI) were collected and compared according to pathogen isolates. There were no statistically significant clinical or laboratory differences between the different bacteria, except for mean platelet count which was lower in patients with *S. pneumoniae* infection (*P* = 0.008).

## DISCUSSION

The retrospective analysis of two cohorts sheds light on the diverse spectrum of LRTI and the alarming trends in antibiotic susceptibility. The first retrospective study included a diverse cohort, revealing distinct pathogen distributions among CA-LRTI, HCA-LRTI, and HA-LRTI patients. Notably, there was a rather high percentage of LRTI caused by Gram-negative bacteria in all study groups, being especially frequent in older patients with multiple comorbidities. On the other hand, as expected, among young and relatively healthy patients, a larger proportion of samples had growth of classic community pathogens (*S. pneumoniae, H. influenza, Bordetella* spp*., Moraxella* spp.). Cases with community-acquired LRTI and no healthcare exposure that were caused by *S. pneumoniae* were more likely to be admitted to an ICU and to die.

Susceptibility to commonly prescribed first-line agents (e.g., penicillin with and without β-lactamase inhibitors) used in the community for the treatment of LRTI was surprisingly low, raising concerns about the effectiveness of empirical treatments.

The limited availability of oral antibiotics for CA-LRTI patients is evident, with susceptibility to penicillin, ampicillin, and macrolides consistently low across all groups. Gram-negative bacteria, predominant in frail elderly patients, displayed resistance to second-generation cephalosporins, penicillins, and β-lactamase inhibitors in more than 50% of cases. The study highlights the inadequacy of certain antibiotics, such as trimethoprim-sulfamethoxazole and second-generation cephalosporins, emphasizing challenges in selecting effective treatments.

The results highlight the importance of optimal treatment selection as many patients with CA-LRTI are treated empirically, and microbiologic data are either received late in the course of treatment or, more commonly, are unavailable altogether. Low-risk patients may fare well with penicillins, macrolides, or second-generation cephalosporins. However, high risk-patients, such as older individuals with multiple comorbidities, may fail initial inadequate treatment and are at an increased risk of complications. The choice of antibiotics is further complicated by potential side effects of fluoroquinolones (7). Furthermore, since CA-LRTIs are very common, resistance to administered antibiotics develops quickly (23), and the ecological impact of antibiotic overuse is a crucial consideration (24). Broader-spectrum antibiotics, such as cefditoren (an oral third-generation cephalosporin), may be a useful alternative for high-risk patients, as they have low rates of reported resistance, even in countries where they are frequently used (25).

Improving diagnostics, including molecular tools for pathogen identification and resistance gene detection, and rapid diagnosis of LRTI caused by viruses offer promising advantages for refining antibiotic choices and avoid unnecessary antibiotic use.

There are some limitations for this study, including the single-center data, retrospective analysis, potential inclusion of non-infected cases, and lack of viral pathogen analysis. In order to assess whether positive respiratory tract cultures were indeed taken from patients with active infection, we screened 50 random samples. Forty-eight samples of these were indeed taken from patients with a respiratory tract infection, supporting the overall validity of our results, but acknowledging that a small minority of tested samples were obtained from patients without LRTI. In addition, susceptibility to cefditoren was assessed with the disk diffusion methods and exact minimum inhibitory concentration (MIC) levels are unavailable. As concordance with susceptibility to ceftriaxone was nearly 100%, we think that this method is accurate enough.

The second part of this study assessed susceptibility to cefditoren and describes a cohort of patients with respiratory tract infections who were recently admitted to the hospital. Respiratory tract bacterial growth samples were taken within 48 h after admission and, therefore, the population reflects severe community-acquired LRTIs, providing valuable insights into bacterial causative agents and their antibiotic susceptibility profiles. Our results highlight a concerning resistance pattern among *S. pneumoniae* and *H. influenzae*. These community pathogens were often resistant to standard penicillin-G doses. Adjusting penicillin doses may address resistance in some *S. pneumoniae* strains but remains inadequate for Gram-negative bacteria or *S. aureus* infections. Cefditoren represents an attractive option for severe LRTIs in the community. Its broad coverage against *S. pneumoniae*, *Haemophilus* spp., and Gram-negative bacteria is comparable to fluoroquinolones and superior to second-generation cephalosporins. These results are very similar to susceptibility patterns in Spain where the drug is used extensively (25). Furthermore, cefditoren has been shown not to lead to increased resistance over the years (26). Transitioning from intravenous cephalosporins to oral cefditoren emerges as an effective outpatient treatment option, aligning with the need of reducing hospital stays and associated costs. The comparable susceptibility rates of ceftriaxone and cefditoren support the feasibility of this sequential strategy. Further research with larger samples would be appropriate.

In summary, these data demonstrate that the choice of antibiotic treatment for LRTI is complicated by high prevalence of resistant bacteria, surprisingly common LRTI caused by Gram-negative bacteria, similarities in clinical features among different causative bacteria, and the scarce microbiologic diagnostics performed in the community. The high rates of drug resistance to penicillin and second generation cephalosporins in hospitalized patients with severe community-acquired LRTI suggest that for selected high-risk patients’ initial coverage with broader spectrum antibiotics such as cefditoren or fluoroquinolones may be appropriate, however, such an approach is yet to be studied prospectively, and could contribute to further resistance if not carefully managed. Improving diagnostics would probably be the best way to optimize antibiotic use.

### Key points

Gram-negative bacteria commonly cause severe respiratory tract infections in elderly patients. Classic community pathogens are more common among younger patients. For high-risk patients, the choice of oral antibiotics is limited as resistance to penicillins and second-generation cephalosporins is common.

## Data Availability

The data sets used and/or analyzed during the current study are available from the corresponding author on reasonable request.

## SUPPLEMENTAL MATERIAL

Figures S1 and S2 spectrum.01422-24-s0001.docx Frequency of bacteria isolated in respiratory tract cultures taken from patients by age group and Charlson Comorbidity Index.

ASM does not own the copyrights to Supplemental Material that may be linked to, or accessed through, an article. The authors have granted ASM a non-exclusive, world-wide license to publish the Supplemental Material files. Please contact the corresponding author directly for reuse.
