# Supplementary material for: Antibiotic susceptibility of pathogens isolated in respiratory tract samples of recently hospitalized patients
Source: Microbiol Spectr. 2025 Feb 5;13(3):e01422-24. doi: 10.1128/spectrum.01422-24 (PMC11878021; doi:10.1128/spectrum.01422-24)
Supplement: Figures S1 and S2 — Frequency of bacteria isolated in respiratory tract cultures taken from patients by age group and Charlson Comorbidity Index. [file spectrum.01422-24-s0001.docx]

Susceptibility of bacteria isolated from patients with respiratory tract infections – supplements.

Figure S1: Frequency* of bacteria isolated in respiratory tract cultures taken from hospitalized patients by age groups

CA-LRTI, Community-acquired lower respiratory tract infection with no healthcare exposure, consist of samples taken within 0-2 days of hospital admission; HCA-LRTI, Community-acquired LRTI with healthcare exposure, consist of samples taken within 0-2 days of hospital admission and with recent healthcare exposure; HA-LRTI, LRTI diagnosed 3-7 days after admission, consist of samples taken within 3-7 days of hospital admission.

*The numbers within the bars represent absolute number of positive cultures

Figure S2: Frequency* of bacteria isolated in respiratory tract cultures taken from patients by Charlson Comorbidity Index

CA-LRTI, Community-acquired lower respiratory tract infection with no healthcare exposure, consist of samples taken within 0-2 days of hospital admission; HCA-LRTI, Community-acquired LRTI with healthcare exposure, consist of samples taken within 0-2 days of hospital admission and with recent healthcare exposure; HA-LRTI, LRTI diagnosed 3-7 days after admission, consist of samples taken within 3-7 days of hospital admission.

*The numbers within the bars represent absolute number of positive cultures
